# Effects of an High-Fat Diet Enriched in Lard or in Fish Oil on the Hypothalamic Amp-Activated Protein Kinase and Inflammatory Mediators

**DOI:** 10.3389/fncel.2016.00150

**Published:** 2016-06-09

**Authors:** Emanuela Viggiano, Maria Pina Mollica, Lillà Lionetti, Gina Cavaliere, Giovanna Trinchese, Chiara De Filippo, Sergio Chieffi, Marcello Gaita, Antonio Barletta, Bruno De Luca, Marianna Crispino, Marcellino Monda

**Affiliations:** ^1^Department of Experimental Medicine-Section of Human Physiology, Second University of NaplesNaples, Italy; ^2^Department of Medicine, University of PadovaPadua, Italy; ^3^Department of Biology, University of Naples Federico IINaples, Italy

**Keywords:** high fat diet, ω3-PUFA, AMPK, inflammation, oxidative stress

## Abstract

The high fat diet (HFD) rich in lard induces obesity, inflammation and oxidative stress, and the deregulation of hypothalamic nuclei plays an important role in this mechanism. One important factor involved in the food intake and inflammation is adenosine monophosphate-dependent kinase (AMPK), a serine/threonine kinase activated by phosphorylation. Omega (ω)3-polyunsaturated fatty acids (PUFA) are dietary compounds known to attenuate the obesity-related diseases, although the molecular mechanisms underlying their actions in the hypothalamus are not completely understood. We hypothesized that the beneficial effects of PUFA may be mediated by AMPK in the hypothalamus. To this aim, rats were fed a control diet (CD), or isocaloric HFD containing either fish oil (FD; rich in ω3-PUFA) or lard for 6 weeks, and the activation of AMPK, inflammatory state (IKKβ, TNF-α) and oxidative stress were analyzed in the hypothalamus. In addition, we also studied serum lipid profile, homeostatic model assessment (HOMA) index, and pro-inflammatory parameters. Our results showed, at the hypothalamic level of LD-fed rats, an increase of AMPK activation, inflammation and oxidative stress, while no modifications were detected in FD-fed animals compared to CD. In addition body weight gain, serum lipid profile, pro-inflammatory parameters and insulin resistance were reduced in FD animals compared to LD. In conclusion, our data indicate that the substitution of saturated by unsaturated fatty acids in the diet has beneficial effects on modulation of hypothalamic inflammation and function in obesity, underlying, at hypothalamic level, the interaction among insulin and/or leptin resistance, AMPK activation and hyperphagia.

## Introduction

Obesity and diabetes are major causes of morbidity and mortality in the western world, and may lead to inflammatory responses and oxidative stress in the peripheral tissues (Hotamisligil, 2006). Several data indicate that overnutrition-associated diseases are also linked to inflammation and increased levels of reactive oxygen species (ROS) in the brain (Cai, 2009). Hypothalamus is the brain region responsible of a variety of metabolic regulations and many overnutrition-related diseases appear to be etiologically related to the deregulations of hypothalamic neurons that are very vulnerable to the nutritional oxidative stress and inflammation (Cai, 2009). It has been demonstrated that overnutrition can activate IKKβ/NF-κB in the mediobasal region of the hypothalamus, which is the area sensing nutrition status and regulating metabolism (Zhang et al., 2008). IKKβ/NF-κB is a master-switch and central regulator of innate immunity and related functions. Some studies have suggested that IKKβ/NF-κB, as mediator of a metabolic inflammation, may represent the link between overnutrition and the dysfunctions of hypothalamic signaling that cause obesity and associated problems (Zhang et al., 2008).

One of the potent counter-regulator of inflammatory signaling pathways is adenosine monophosphate-dependent kinase (AMPK; Salt and Palmer, 2012; Hernández-Aguilera et al., 2013). AMPK, an evolutionary conserved serine/threonine kinase, is a heterotrimeric complex formed by a catalytic α subunit and regulatory β and γ subunits. The phosphorylation of the α subunit, at Thr172, turns AMPK into the activated form pAMPK. AMPK is a sensor of the cellular energy status that, when activated by metabolic stress, is able to maintain cellular energy homeostasis by turning on catabolic pathways (Hardie, 2014).

Several studies have demonstrated that AMPK in the hyphotalamus regulates food intake and that its activity is modulated by several hormones such as leptin, adiponectin and insulin. Since these hormones are involved in the control of body weight and glucose and lipid metabolism, these findings implied that AMPK might serve as a downstream effector of leptin and insulin and thereby play a role as a signaling molecule during hormon-induced metabolic responses. Whereas leptin inhibits AMPK activity in the arcuate and in the paraventricular nuclei of hypotalamus, insulin is known to inhibit AMPK activity in the lateral, ventromedial, and dorsomedial hypothalamic regions (Minokoshi et al., 2004). All these brain regions are involved in the control of food intake (Monda et al., 1993; Viggiano et al., 2006) and in energy expenditure (Monda et al., 1996; Messina et al., 2013).

The ω-3 polyunsaturated fatty acids (PUFA), docosahexaenoic acid (DHA) and eicosapentaenoic acid (EPA), are dietary compounds that are intensively studied as potent anti-inflammatory products, able to reduce the risk of insulin resistance and ameliorate obesity-associated disorders affecting hormonal control and modulating AMPK activity (Xue et al., 2012; Martínez-Fernández et al., 2015). Recently, we have demonstrated that the replacement of lard, rich in saturated fatty acids (SFA), with fish oil (rich in ω-3 PUFA) in high-fat diet (HFD) is able to limit the development of systemic and tissue inflammation, hepatic steatosis and to attenuate insulin resistance (Lionetti et al., 2014a,b; Cavaliere et al., 2016).

Here, we have tested, at hypothalamic level, the effect of substitution of saturated by unsaturated fatty acid on AMPK, activated AMPK (pAMPK at Thr 172), IKKβ, inflammation and oxidative stress. Moreover, we have also analyzed, in the same animals, the involvement of insulin, leptin and inflammatory parameters in the modulation of AMPK.

## Materials and Methods

All chemicals were purchased by Sigma–Aldrich (St. Louis, MO, USA), unless otherwise specified. Young male Wistar rats (60 days old; 345 ± 7 g; Charles River, Calco, Lecco, Italy) were individually caged in a temperature-controlled room and exposed to a daily 12/12 h light/dark cycle with free access to chow diet and drinking water. Rats were divided into three experimental groups (*n* = 8) according to a different 6 weeks dietary regimen: the first group (control diet, CD) received a standard diet (10.6%fat J/J); the second group (LD) received the HFD rich in lard (40% fat J/J); and the third group (FD) received the HFD rich in fish oil (40% fat J/J). The composition of all dietary regimens is reported in Table 1.

**Table 1:** **Diet composition**.

	Control diet	HFD rich in lard (g/100 g diet)	HFD rich in fish oil (g/100 g diet)
Standard feed	100	51.30	51.30
Casein^a^ g	–	9.25	9.25
Lard g	–	21.80	–
Fish oil^b^ g	–	–	21.80
Sunflower oil g	–	1.24	1.24
AIN 76 mineral mix^c^ g	–	1.46	1.46
AIN 76 vitamin mix^d^ g	–	0.42	0.42
Choline bitartrate	–	0.08	0.08
Methionine g	–	0.12	0.12
Energy density kJ/g diet	15.88	20	20
Protein %	29	29	29
Lipid %	10.60	40	40
Carbohydrate %	60.40	31	31

*^a^Purified high-nitrogen casein containing 88% protein, ^b^Fish oil = Cod liver Oil, ^c^American Institute of Nutrition (1977), ^d^American Institute of Nutrition (1980)*.

Throughout the experimental period, body weights and food intakes were monitored daily to calculate the body-weight gain and the gross energy intake. Spilled food was collected and compensated in readjusting the calculation of food intake. Gross energy density for standard or HFD (15.8, or 20 kJ/g, respectively) was determined by a bomb calorimeter (Parr adiabatic calorimeter, Parr Istrumentes Co, Moline, IL, USA).

Another set of CD, LD and FD animals (*n* = 5 per group) at 6 weeks of treatment, were injected i.p. with insulin (homolog rapid-acting, 10 units/kg body wt; Novartis, Basel, Switzerland).

At the end of the experimental treatments, the rats were anesthesized by i.p. injection of chloral hydrate (40 mg/100 g body weight), decapitated with a guillotine, and the blood was taken from the inferior cava vein. The hypothalamus was quickly dissected from the brain and transferred in the appropriate buffer. All the samples that were not immediately used were stored at −80°C.

### Serum Parameters

The serum levels of cholesterol, triglycerides, NEFA and glucose were measured with standard procedures. The serum levels of insulin (Mercodia AB, Uppsala, Sweden), TNF-α (Biovendor R&D, Brno, Czech Republic), adiponectin and leptin (B-Bridge International Mountain View, CA, USA) were measured using commercially available ELISA kits.

### Lipid Peroxidation Assay

To determine the lipid peroxidation in hypothalamic homogenate, the level of malondialdehyde (MDA) was measured using the thiobarbituric acid reaction (TBAR) method. MDA reacts with thiobarbituric acid (TBA) to form a pink chromogen that is detected at the wavelength of 532. MDA values were expressed as nanomoles per milligram of brain protein (Lu et al., 2009).

### Redox Status and Nuclear Factor Erythroid 2-Related Factor (Nrf2) Activated Enzymes Activities

Reduced glutathione (γ-L-Glutamyl-L-Cysteinyl-Glycine, GSH) and oxidized glutathione (γ-glutamyl-L-cyteinylglycine disulfide, GSSG) concentrations in the hypothalamus were measured using the dithionitrobenzoic acid (DTNB)-GSSG reductase recycling assay (Bergamo et al., 2007); the GSH/GSSG ratio was used as an oxidative stress marker. The enzymatic activities of glutathione S-transferase (GST) and NAD(P)H-quinone oxidoreductase (NQO1) were evaluated spectrophotometrically in brain cytoplasmic extracts with standard protocols (Benson et al., 1980; Levine et al., 1990).

### Western Blot

The hypothalamus was homogenized in the lysis buffer (10 mM HEPES, 10 mM KCl, 1.5 mM MgCl_2_, 12% glycerol, 0.5 mM DTT, 0.1 mM EGTA) with a cocktail of protease inhibitors (Sigma Aldrich). Proteins (20 or 40 μg/lane) were separated on 12% SDS-PAGE and transferred to nitrocellulose membranes. The blots were incubated with AMPKα rabbit monoclonal antibody (Cell Signaling Technology; dil 1:1000), pAMPKα (Thr172) rabbit monoclonal antibody (Cell Signaling Technology; dil 1:1000), IKK-β rabbit monoclonal antibody (Abcam; dil 1:500) or α-tubulin mouse antibody (Sigma Aldrich; dil 1:1000) overnight at 4°C and then with secondary antibody against rabbit or mouse IgG (Promega; dil 1:2500) for 1 h at RT. The signals were visualized with the ECL system (Pierce). The expression level of α-tubulin was used to normalize the data.

### Statistical Analysis

Statistical analyses were carried out using SPSS 13.0 (SPSS Inc., Chicago, IL, USA). ANCOVA or one way ANOVA followed by Tukey’s *post hoc* test was used to evaluate differences between the groups. *P* values smaller than 0.05 were considered statistically significant.

### Ethics Statement

Procedures involving animals and their care were conducted in conformity with international and national law and policies (EU Directive 2010/63/EU for animal experiments, ARRIVE guidelines and the Basel declaration including the 3R concept). The procedures reported here were approved by the Institutional Committee on the Ethics of Animal Experiments (CSV) of the University of Naples Federico II and by the Ministero della Salute****.

## Results

### Effect of High Fat Diet Enriched in Lard or Fish Oil on Body Weight Gain and Energy Intake

**Figure 1:**
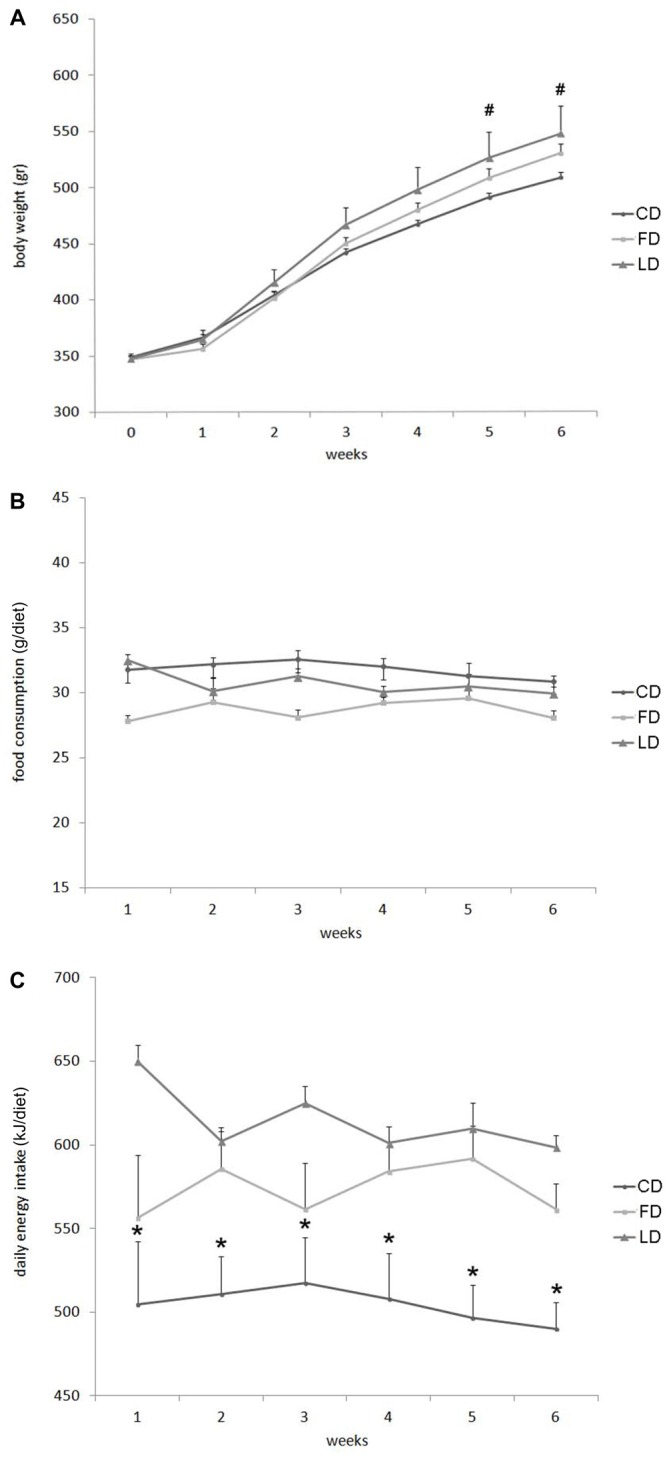
**Effect of high fat diet enriched in lard or in fish oil on body weight, food consumption and energy intake.** Body weight **(A)**, food consumption **(B)** and daily energy intake **(C)** during 6 weeks of treatment with control diet (CD), fish oil enriched diet (FD) or lard enriched diet (LD). Values are expressed as Mean ± SEM. ^#^*P* < 0.05 compared to the CD group; *P < 0.05 compared to the other groups.

Figure 1 reports the changes in body weight, daily food consumption (g/day) and energy intake in LD or FD fed rats compared to CD at 1–6 weeks of treatment. At each time point, the body weight was higher in the LD compared to CD, but the difference was statistically significant only after 5 and 6 weeks of treatment (Figure 1A). On the other hand, the three groups of rats did not show any difference in the daily food consumption (g/diet, Figure 1B). whereas the energy intake (kJ/diet) was significantly higher in the LD and FD groups compared to the CD group at each time point (Figure 1C).

### Effect of High Fat Diet Enriched in Lard or Fish Oil on Plasma Lipids, Hormones and TNF-α

**Figure 2:**
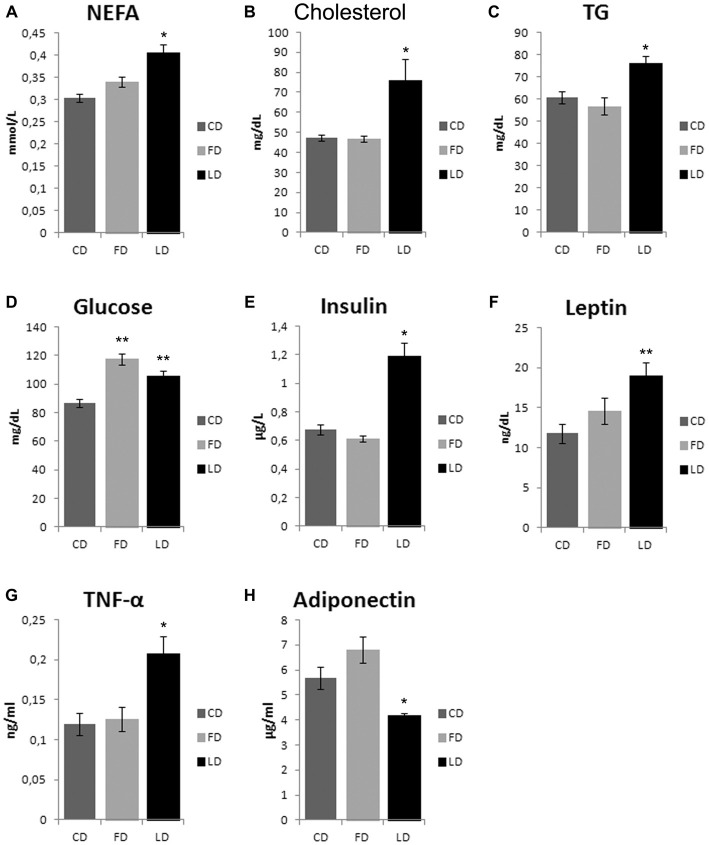
**Effect of high fat diet enriched in lard or in fish oil on plasma lipids, glycemia, hormones and inflammatory index.** Serum levels of non-esterified fatty acid (NEFA, **A**), cholesterol **(B)**, triglycerides (TG, **C**), glucose **(D)** insulin **(E)**, leptin **(F)**, TNF-α **(G)** and adiponectin **(H)**, in animals treated for 6 weeks with control diet (CD), fish oil enriched diet (FD) or lard enriched diet (LD). Values are expressed as Mean ± SEM. *P < 0.05 compared to CD and FD; **P < 0.05 compared to CD.

As shown in Figure 2, the HFD enriched with fish oil for 6 weeks increased significantly basal glycemia compared to the CD (*P* < 0.05), while the other parameters (non-esterified fatty acid (NEFA), cholesterol, TG, insulin, leptin, TNF-α and adiponectin) did not change compared to CD, although they were significantly different from LD group.

### Effect of the High Fat Diet Enriched in Lard or Fish Oil on the Phosphorylation of the Hypothalamic AMPK

**Figure 3:**
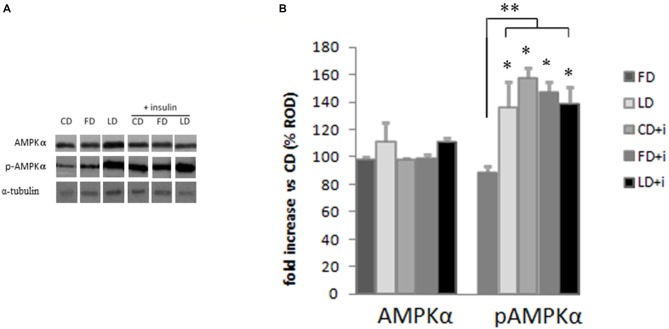
**Effect of high fat diet enriched in lard or in fish oil on hypothalamic AMPK or pAMPK in rats with or without insuline injection. (A)** Representative blots probed with antibodies against p-AMPK (phosphorylated at Thr172), AMPK and α-tubulin. Samples were obtained by hypothalamus of animals treated for 6 weeks with control diet (CD), fish oil enriched diet (FD) or lard enriched diet (LD). Some of the animals were injected with insulin (+ insulin). **(B)** Quantitation of protein levels of AMPK and pAMPK normalized to the α-tubulin by densitometry. The bars show the values (Mean ± SD) expressed as percentage of CD group as Relative Optical Density (ROD). *P < 0.05 compared to CD and FD. **P < 0.05 compared to FD.

The HFD (enriched in lard or fish oil) did not modify the expression of AMPK in the hypothalamus as judged by the results of Western blot analyses (Figure 3). To investigate the effects of the HFD on the activity of this protein, we also analyzed the expression level of pAMPK (the phosphorylated active form of AMPK) and we did not observe any difference between FD and CD groups (Figure 3), indicating that the fish oil-enriched diet did not change the hypothalamic expression level of AMPK and its activation level. Interestingly however, the pAMPKα expression level significantly increased in the LD group compared to FD group (Figure 3).

To evaluate the involvement of insulin sensitivity in the LD-dependent increase of pAMPK, the three groups of animals were intraperitoneally injected with insulin. The insulin did not modify the AMPK expression level in the hypothalamus. Interestingly, the insulin in CD and FD groups increased significantly the pAMPK level in the hypothalamus compared with the corresponding groups without insulin treatment. On the other hand the pAMPK expression level did not change in LD group treated with insulin (Figure 3).

### Effect of High Fat Diet Enriched in Lard or Fish Oil on Hypothalamic IKKβ

To investigate the molecular mechanisms underlying the HFD-dependent activation of AMPK we analyzed the expression level of IKKβ (activator of NFKB pathway) in the hypothalamus of treated animals. We did not observe significant changes of this mediator in LD or FD groups compared to CD, suggesting that the mechanism induced by LD-enriched diet and leading to the activation of hypothalamic AMPK is independent from IKKβ (data not shown).

### Effect of High Fat Diet Enriched in Lard or Fish Oil on Inflammation and Oxidative Stress in the Hypothalamus

**Figure 4:**
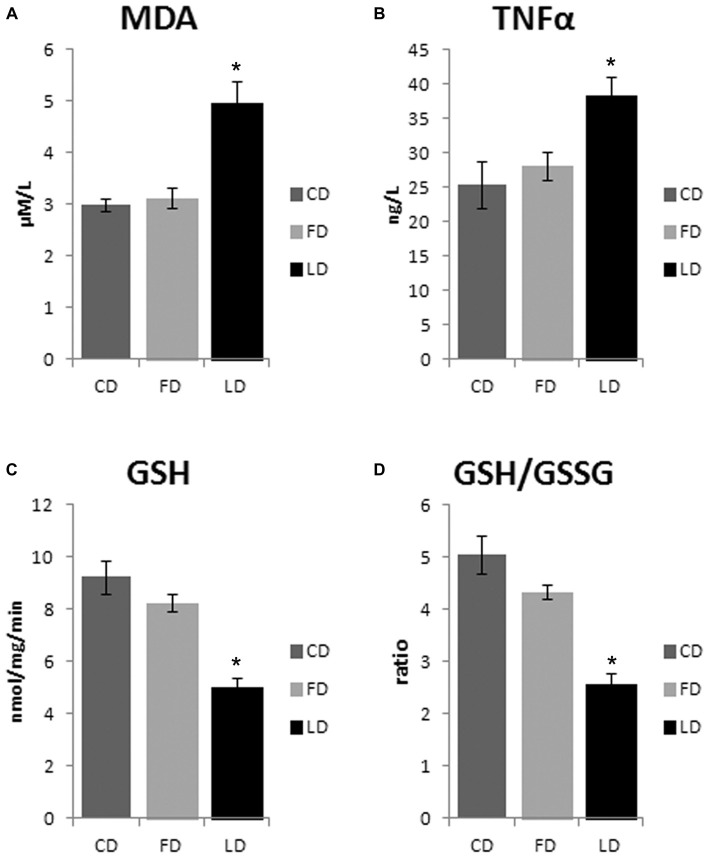
**Effect of high fat diet enriched in lard or in fish oil on hypothalamic oxidative stress.** Hypothalamic levels of malondialdehyde (MDA, **A**), TNF-α **(B)**, reduced glutathione (GSH, **C**) and ratio reduced/oxidized glutathione (GSH/GSSG, **D**) in animals treated for 6 weeks with control diet (CD), fish oil enriched diet (FD) or lard enriched diet (LD). Values are expressed as Mean ± SEM. *P < 0.05 compared to CD and FD.

The HFD enriched in LD causes an increase of oxidative stress in the hypothalamus as demonstrated by the significant increase of MDA (*P* < 0.01) and TNFα (*P* < 0.05) and the significant decrease of GSH and GSH/GSSG (*P* < 0.01) in LD compared to FD and CD groups. On the other hand, the HFD enriched in fish oil did not affect these parameters (that are similar to control) demonstrating beneficial effects of fish oil on oxidative stress in the hypothalamus (Figure 4). In addition, the negligible differences in GST and NQO1 activities in LD and FD groups compared to CD indicated that the Nrf2 pathway is not involved in the HFD-mediated modulation of redox status (data not shown).

## Discussion

The present study aims to evaluate the effect of the nutritional substitution of saturated by unsaturated fatty acids on the modulation of hypothalamic inflammation in obesity. Our data confirmed that the extent of HFD harmful effects depends on the type of fat included in the diet and in particular, the HFDs rich in poly-unsaturated fatty acids (fish oil) are less deleterious than those rich in saturated fat (lard).

Rats fed for 6 weeks with HFD enriched in lard or fish oil showed an increase in body weight when compared to a control group. On the other hand, in previous studies, using the same experimental paradigm, we found in FD group a significant decreased in body fat mass compared to LD group (Cavaliere et al., 2016). In addition, we also showed that the increase in body weight was not associated with an increase in food intake, but with an increase in energy intake, in agreement with several previous studies (Cavaliere et al., 2016). In this context, it is worth to notice that rodents are considered hyperphagic if the energy intake increases even in conditions of normal food intake (Ghibaudi et al., 2002; Woods et al., 2003; Huang et al., 2004).

To verify the hypothesis that the increase in energy intake may depend on activation of the AMPK pathway in the hypothalamus, that is strongly involved in the control of food intake (Minokoshi et al., 2004), we studied the effect of a HFD on the hypothalamic AMPK expression. Although the hypothalamic expression of AMPK was not modified by diet, the level of the active phosphorylated form of the enzyme (pAMPK, phosphorylated at Thr 172) was significantly increased in LD group, suggesting that the administration of this diet leads to the activation, at hypothalamic level, of a key enzyme regulating the food intake. This effect was observed exclusively with LD diet suggesting that the type of fats affects pAMPK expression. The activation of AMPK in LD group is independent from IKKβ, an inhibitor of NF-κB, since the IKKβ expression level did not change with the administration of HFD.

We also observed that HFD rich in lard, unlike HFD rich in fish oil, affects the insulin, leptin and adiponectin levels. Therefore, a possible mechanism underlying the AMPK activation in the hypothalamus of LD animals may involve orexinergic or anorexinergic molecules. Indeed, several orexinergic and anorexinergic molecules are known to modify the AMPK activity in the hypothalamus. In particular, glucose, leptin and insulin inhibit AMPK (Minokoshi et al., 2004; Kim and Lee, 2005), and the HFD induces resistance to leptin (Martin et al., 2006) and insulin (Lichtenstein and Schwab, 2000; Woods et al., 2003; Riccardi et al., 2004). Leptin and insulin are present in the hypothalamic neurons that are involved in body weight regulation, and the loss of leptin and insulin in the hypothalamus can promote obesity and type 2 diabetes (Morton and Schwartz, 2011). In the present study, we have shown that the insulin injection increased hypothalamic expression of pAMPKα in the CD group and in the FD group, but it had no effect in the LD group. A possible explanation could be that the increased hypothalamic expression of pAMPK, following insulin injection in the CD group and in the FD group, was mediated by hypoglycemia in agreement with previous findings that hypoglycemia activates AMPK (Han et al., 2005). The LD group instead, might have developed insulin resistance, as previously reported (Xu et al., 2003; Hancock et al., 2008; Cavaliere et al., 2016), resulting therefore in the lack of hypoglycemic effect of exogenous insulin, and in turn, no further increase in hypothalamic pAMPK expression. Central and peripheral insulin resistance could depend on inflammatory molecules. In fact, overnutrition can induce inflammatory responses in peripheral tissues and in the hypothalamus, resulting in a defective control of food intake and energy expenditure (Minokoshi et al., 2004; De Souza et al., 2005). In particular, hypothalamic inflammation affects whole body energy homeostasis mostly by controlling neural inputs to specific organs (Thaler et al., 2010, 2013). Here, we found in FD group a reduction in the hypothalamic inflammation (indicated by decreased TNFα level) and oxidative stress (indicated by decreased MDA level). Nonetheless, the modulation of inflammatory state and redox state does not depend on Nrf2 pathway since GST and NQO1 activities do not change in the three groups of rats. Further studies are necessary to investigate on other molecules that may mediate the anti-inflammatory and anti-oxidant effects of polyunsaturated fatty acid. The reduction of hypothalamic inflammation in obesity corrects simultaneously feeding, thermogenesis and metabolic disarrangements, placing this phenomenon in a central position in the pathogenesis of obesity (De Souza et al., 2005; Milanski et al., 2009).

Our data confirm that chronic overnutrition leads to hypothalamic dysregulation and to the development of overnutrition-related diseases. Interestingly, the substitution of saturated by unsaturated fatty acids in the diet has beneficial effects on modulation of hypothalamic inflammation and function in obesity, underlying, at hypotalamic level, the interaction among insulin and/or leptin resistance, AMPK activation and hyperphagia.

## Author Contributions

EV and MPM conceived the original idea, designed and supervised the whole study; MPM, EV, GC, GT, CDeF and MG performed the experiments; MPM and EV analyzed and interpreted data; EV, MPM, LL, SC, AB, BDeL MC and MM critically revised the manuscript for intellectual content; EV, MPM, MM and MC wrote the article.

## Conflict of Interest Statement

The authors declare that the research was conducted in the absence of any commercial or financial relationships that could be construed as a potential conflict of interest.
