# Supplementary figures and images for: Effects of an High-Fat Diet Enriched in Lard or in Fish Oil on the Hypothalamic Amp-Activated Protein Kinase and Inflammatory Mediators
Source: Front Cell Neurosci. 2016 Jun 9;10:150. doi: 10.3389/fncel.2016.00150 (PMC4899473; doi:10.3389/fncel.2016.00150)

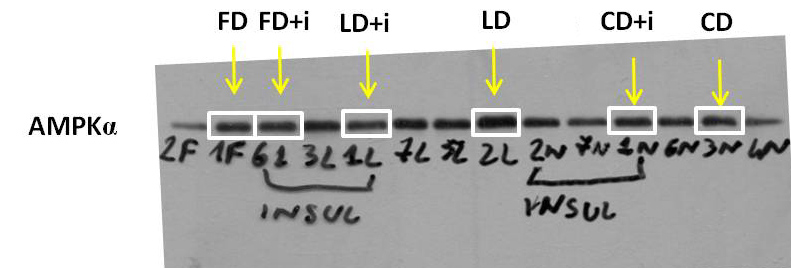

Supplement: Supplementary file 1 [file Image_1.jpeg]

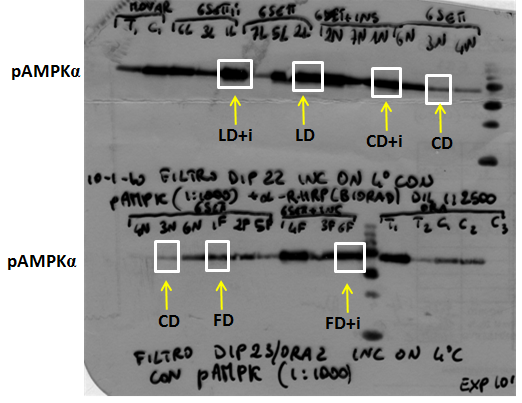

Supplement: Supplementary file 2 [file Image_2.tif]
